# Recent increase in atypical presentations of invasive meningococcal disease in France

**DOI:** 10.1186/s12879-024-09547-y

**Published:** 2024-06-26

**Authors:** Samy Taha, Ala-Eddine Deghmane, Muhamed-Kheir Taha

**Affiliations:** Invasive Bacterial Infections Unit and National Reference Centre for Meningococci and Haemophilus influenzae, Institut Pasteur, Université Paris Cité, 28 rue du Dr Roux, Paris cedex 15, 75724 France

**Keywords:** Invasive meningococcal disease, Epidemiology, COVID-19, *Neisseria meningitidis*, Clinical presentation

## Abstract

**Background:**

Invasive meningococcal disease (IMD) cases declined upon the implementation of non-pharmaceutical interventions (NPI) (social distancing and mask wearing) to control the COVID-19 pandemic but rebounded in 2022 in numbers with genotypical changes of the strains. We explored here associated modifications in the clinical presentations of IMD.

**Methods:**

We conducted a retrospective descriptive study using the Database of the French National Reference Centre for meningococci and *Haemophilus influnezae* for IMD cases between 2015 and 2022. We scored serogroups, sex, age groups, clinical presentations and clonal complexes of the corresponding patients and isolates.

**Findings:**

Non-meningeal forms of IMD increased significantly upon easing of NPI, such as bacteremic meningococcal pneumonia and bacteremic abdominal forms. They represented 6% and 8% of all IMD forms and were significantly linked to serogroups Y and W respectively, to older adults for bacteremic pneumonia and to young adults for bacteremic abdominal presentations. These forms were significantly associated with more early mortality and clonal complexes 23, 11 and 9316.

**Interpretation:**

The increase in atypical IMD forms may lead to higher burden of IMD due to delayed diagnosis and management. Updating prevention may be needed through by adapting the current vaccination strategies to epidemiological changes.

**Supplementary Information:**

The online version contains supplementary material available at 10.1186/s12879-024-09547-y.

## Introduction

*Neisseria meningitidis* (Nm) is usually carried asymptomatically in the nasopharynx of approximately 10% of the general population [1]. However, invasive isolates of *N. meningitidis* can cross the respiratory epithelial barrier and infect the bloodstream and lead to severe, rapidly evolving systemic disease called invasive meningococcal disease (IMD). IMD is dominated by bacteremia and meningitis that represented 37% and 53% of all the cases in Europe in 2018 respectively [2]. Other non-meningeal presentations (other than bacteremia) of IMD were rarely described and represented 10% of IMD cases in Europe in 2018 [2]. They are linked to the hematogenous spread of meningococci after crossing the respiratory epithelial barrier and their extra-meningeal localization [3]. However, the frequency of these forms may be underestimated, as they can also co-exist with usual presentations such as Bacteremia and meningitis [4].

Recent epidemiological changes and the emergence of particular strains significantly more linked to those atypical presentations raised the need to inform on unusual/atypical clinical forms of IMD [5]. For example, abdominal presentations, which are defined by abdominal pain, gastroenteritis, diarrhea or peritonitis associated to the isolation of Nm in a sterile site, were reported in only 1% of IMD cases in France between 1999 and 2002 [6]. However, these abdominal presentations increased and were associated with the South America-UK W/cc11 strain and its derivatives reaching 17% of cases of IMD due to this genotype between 2014 and 2016 with a higher fatality rate than the usual clinical presentations [7–9].

If untreated, IMD is almost always fatal, and even treated it still shows a high fatality rate of 10% and up to 20% of survivors suffer permanent disabling sequelae that impair their quality of life [10]. Recognition of IMD at an early stage is still difficult as early symptoms and signs are usually non-specific, particularly with atypical forms, which may delay the management and increase the burden of the disease [11].

Upon the implementation of COVID-19 control measures (such as social distancing, mask wearing*)*, the number of IMD cases per year declined sharply in 2020 across all serogroups and age groups [12]. This decline continued in 2021 until September 2022 but showed a significant rebound since then and continued in the beginning of 2023 to reach higher levels than the pre-COVID-19 period, with profound epidemiologic and genotypic changes: a more important increase in IMD cases due to serogroups W and Y but a decrease of the historical hyperinvasive genotypes such as the clonal complex cc11 that used to account for the majority of serogroup W cases in France since the beginning of the 2010s [12].

The aim of this study is to describe the impact of those epidemiological changes in bacterial isolates on the clinical manifestations of IMD and particularly on unusual forms.

## Methods

### Data sources

Reporting of IMD cases is mandatory and is performed by physicians and laboratories to the regional health agencies according to the French case definition and data are then centralized by Santé Publique France [12]. Additionally, cultured isolates and samples are systematically sent to the National Reference Centre for meningococci and *Haemophilus influenzae* (NRCMHi) for full phenotyping and genotyping, including whole genome sequencing (WGS). Data from both country-wide sources (mandatory reporting and bacteriological characterization) are combined. The completeness of the surveillance system was estimated at > 91% [13]. Sending material to the NRCMHi is accompanied by a standardized clinical record form (available at https://www.pasteur.fr/fr/file/16390/download) that is completed by the clinician and microbiologist at the hospital where patients are admitted. The records include the age and sex of the patient and clinical and biological findings. Typing of the isolates and samples was performed by Multilocus sequence typing (MLST) on primary samples and by whole genome sequencing (WGS) for culture-confirmed cases, as previously described [12, 14].

### Data analysis

Isolates and samples for the 2015–2022 period were extracted from the NRCMHi database. Data were curated for duplicate entries. We used GraphPad PRISM 5.0.1 software for statistical analysis (https://www.graphpad.com/). Chi-square tests were used to compare the observed number of cases to the expected number. The significance level was *P* < 0.05 for single comparisons. This threshold was adjusted using the Bonferroni correction for multiple comparisons. A descriptive analysis of the number of cases, age, sex, serogroup, clinical presentations and clonal complex distribution by year was performed. Age groups (< 1 year, 1–4 years, 5–9 years, 10–14 years, 15–19 years 20–24 years, 25–44 years, 45–64 years and ≥ 65 years) were used according to WHO code list (available at: https://apps.who.int/gho/data/node.searo-metadata.AGEGROUP?lang=en). Genomic analysis was performed using tools available on PubMLST database to define the genotypes (clonal complexes) of Nm isolates [15, 16].

### Definition of the clinical presentations

NPI implementation and their impacts were previously reported [17]. IMD cases are confirmed on the basis of bacterial detection in a normally sterile site [13]. Bacteremia/sepsis presentations were defined by the detection of Nm (by culture and/or PCR) in a blood sample with or without purpura fulminans (extensive ulcero-necrotic purpura) or other purpuric lesions.

Meningeal presentations were defined as the detection of Nm in the cerebrospinal fluid (CSF) and/or in the blood with more than 10 leucocytes in the CSF.

Bacteremic meningococcal pneumonia presentations were defined as the detection of Nm in the blood with respiratory symptoms and radiological images compatible with pneumonia.

Abdominal presentations were defined as the detection of Nm in the blood with acute abdominal pain or diarrhea or gastro-enteritis with diarrhea and vomiting but no detection of other pathogens in stool specimens.

Meningococcal arthritis presentations were defined as the detection of Nm in joint fluid with arthralgia.

Each case was classified for each presentation definition it met. One case could therefore be classified according to multiple presentations. Early mortality was scored and was defined as a death indicated on the clinical records communicated to the NRCMHi by the hospital of admission and covered the period up to the detection of Nm, and therefore corresponds to a death occurring within the first 72 h of the illness.

### Ethics approval

These data were included anonymously in the database after excluding personal data as part of the mission of the National Reference Centre for meningococci and *Haemophilus influenzae* (NRCMHi) for routine surveillance of IMD and isolate identification and typing. The procedure for collecting samples and information was submitted and approved by the CNIL N°1,475,242/2011 (*Commission Nationale de l’Informatique et des Libertés)* and the requirement for consent was waived.

### Role of funding source

The study was funded by the Institut Pasteur and Santé Publique France. Both funders had no role in designing, conducting, analyzing, and writing the study. Study design, data collection, data analysis, data interpretation, and writing of this report were performed by the authors of the study.

## Results

Characteristics of IMD cases between 2015 and 2022 for the number of cases, average age and sex ratio were previously reported in another study [12]. It includes 2,719 cases of IMD between 2015 and 2022 with no significant variation of the average sex ratio and the average median age [12].

### Evolution of the usual clinical presentations between 2015 and 2022

#### By year

**Fig. 1:**
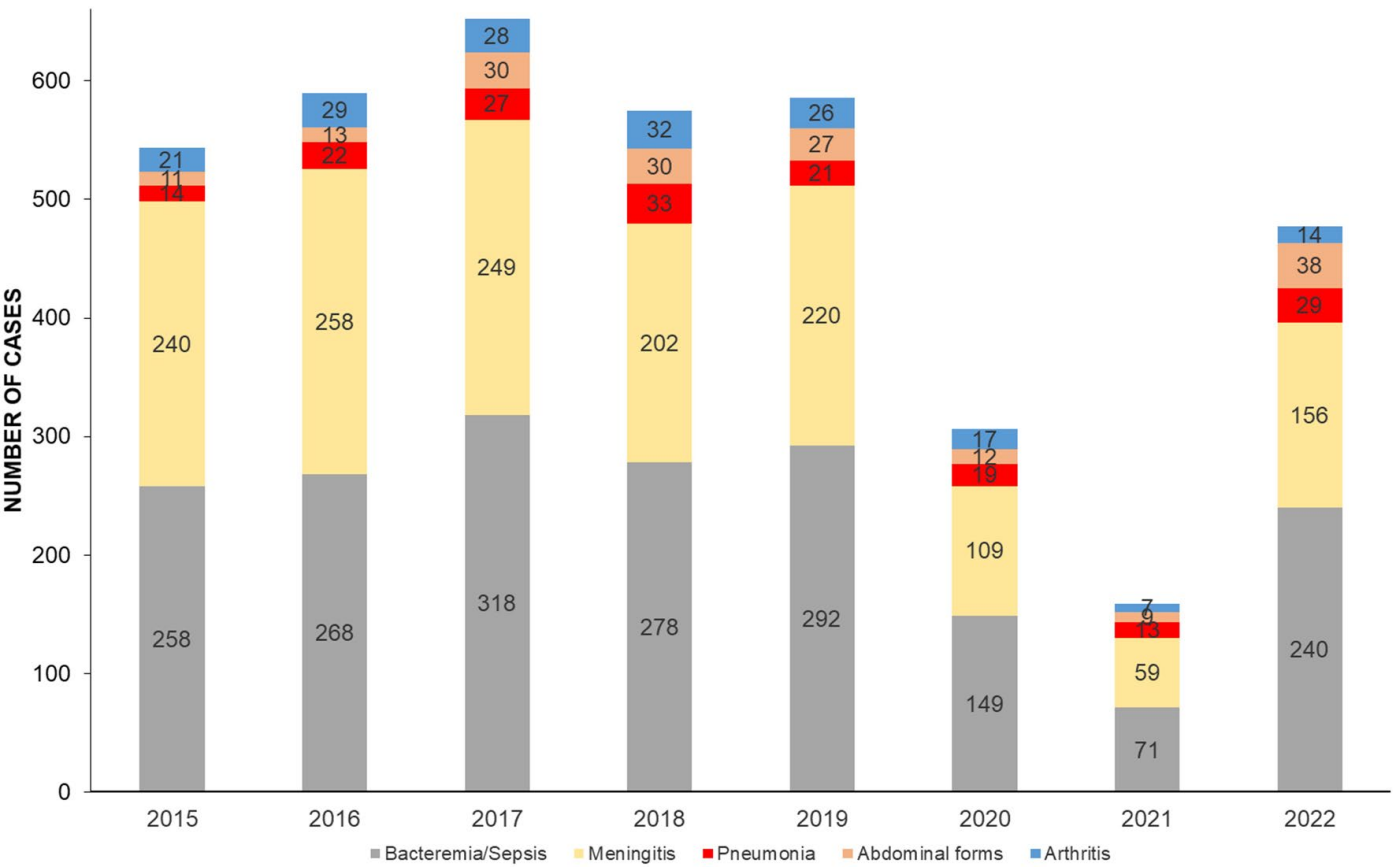
Distribution of clinical presentations of IMD per year in France, 2015–2022

The Bacteremia/sepsis clinical presentation remained the main presentation of IMD during the studied period. Its percentage remained stable during the whole study period, varying non-significantly (*p* = 0.94) between 44.7% (71 cases out of a total of 159 clinical forms in 2021) and 50.3% % (240 cases out of a total of 477 clinical forms in 2022). Meningeal presentation was significantly more frequent in proportion before the COVID-19 pandemic. It decreased from 44.1% (240 cases out of a total of 544 clinical forms in 2015) to 32.7% (in 156 cases out of a total of 477 clinical forms in 2022) but this reduction remained non-significant (*p* = 0.03 with an adjusted threshold at 0.01 for 5 comparisons as described in the data analysis paragraph of the methods section) (Fig. 1).

#### By serogroup

**Fig. 2:**
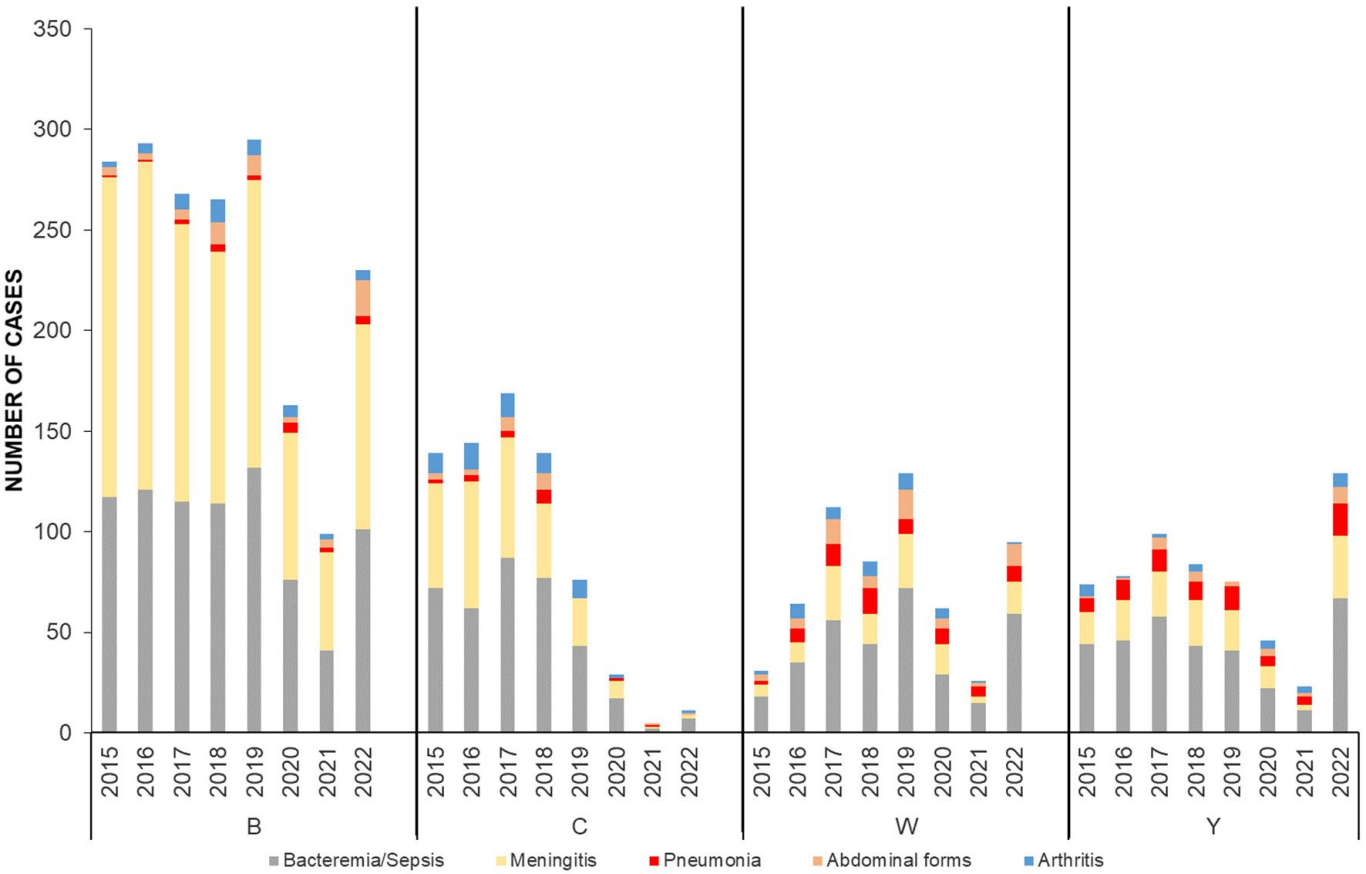
Evolution of IMD clinical presentations by serogroup, France, 2015–2022

Since the 2022 rebound of cases until the end of the study period, bacteremia/sepsis became significantly associated with serogroups W and Y (*p* < 0.0001). Meningeal presentation was significantly associated with serogroup B during the whole study period (*p* < 0.0001) (Fig. 2).

#### By age group

**Fig. 3:**
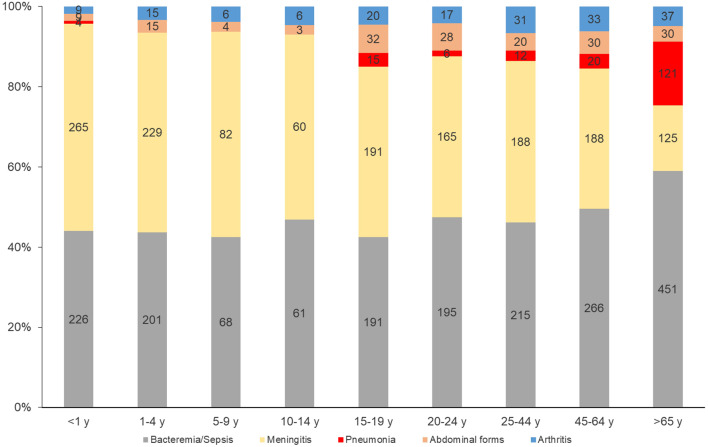
Distribution of clinical presentations by age group, France, 2015–2022. (number of cases are indicated in the bars)

Meningeal forms were more prevalent among the less than 5-year-olds (*p* < 0.0001). Bacteremia/sepsis also was significantly associated with older adults of ≥ 65 years of age (*p* < 0.0001) (Fig. 3).

#### By clonal complex

**Fig. 4:**
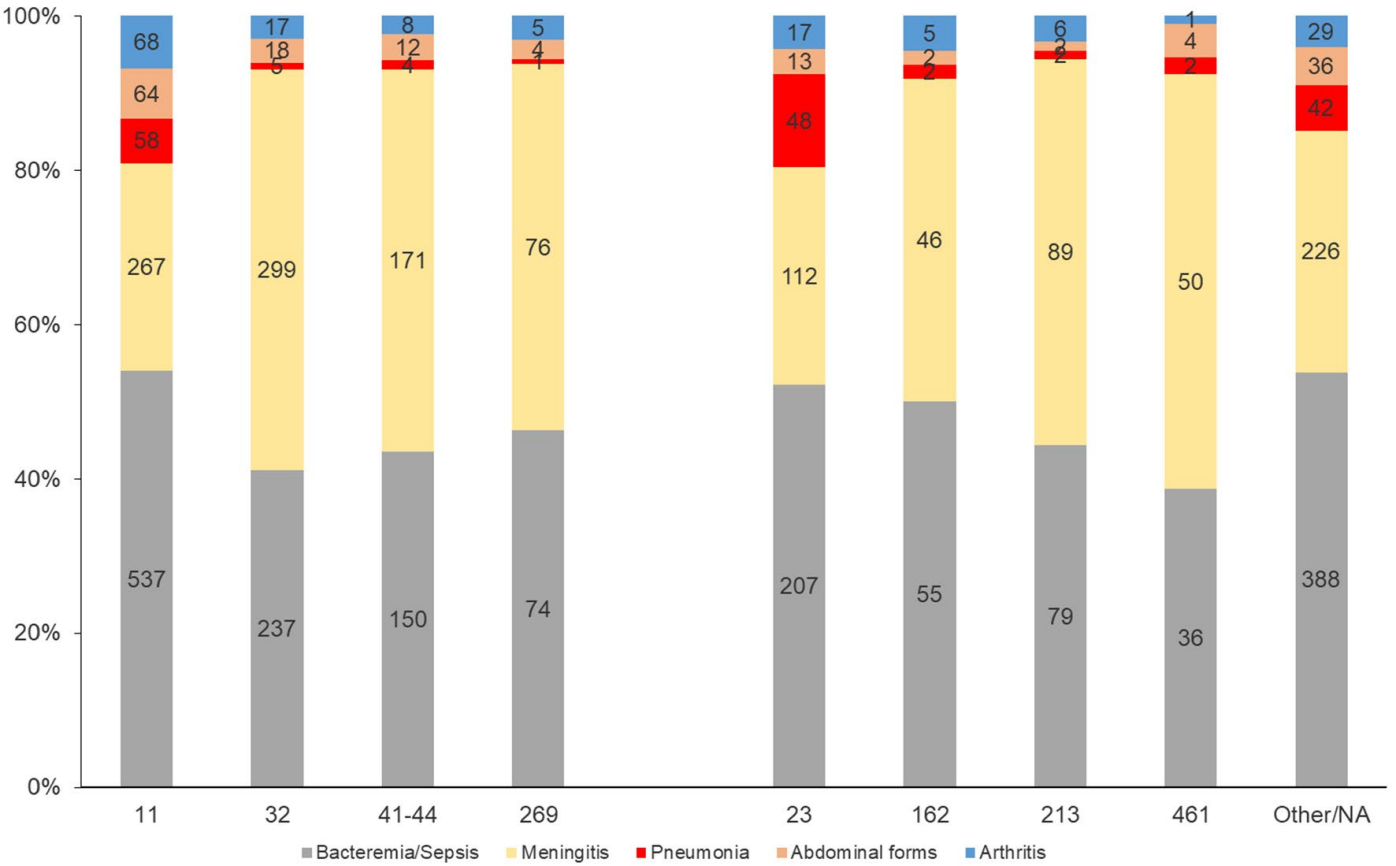
Distribution of clinical presentations by clonal complex, France, 2015–2022. (number of cases are indicated in the bars)

Bacteremia/sepsis was significantly more frequent with isolates of the clonal complex 11 (*p* < 0.0001). However, bacteremia/sepsis was also linked to isolates that were non-assigned to known clonal complexes (*p* < 0.0001). Meningeal presentation was significantly associated with clonal complex 32 (*p* < 0.0001), 41/44 (*p* < 0.0001), and 213 (*p* = 0.001) which were all related to IMD due to serogroup B isolates (IMDB) (Fig. 4).

### Evolution of the atypical clinical presentations between 2015 and 2022

#### By year

There was an increase of bacteremic meningococcal pneumonia increasing from 2.6% of all clinical forms in 2015 up to 8.2% in 2021 and 6.1% in 2022 but these variations remained at the level of significance (*p* = 0.01). Abdominal presentations also significantly increased during the study period and particularly in 2022 (*p* = 0.0001), increasing from 2% in 2015 to 5.7% in 2021 and 8% in 2022. Conversely, meningococcal arthritis did not significantly change during the study period and varied between 5.6% and 2.9%. (Fig. 1).

#### By serogroup

Both bacteremic meningococcal pneumonia and abdominal presentations were significantly associated with serogroups W (*p* < 0.0001). Bacteremic meningococcal pneumonia was also significantly associated with serogroup Y (*p* < 0.0001). Meningococcal arthritis was significantly associated with serogroup C and W (*p* < 0.0001) (Fig. 2).

#### By age group

Bacteremic meningococcal pneumonia was significantly more prevalent among older adults of ≥ 65 years of age (*p* < 0.0001) whereas abdominal presentations were more frequent among the 20–24 years-olds (*p* = 0.005). Meningococcal arthritis wasn’t significantly associated with a specific age group (Fig. 3).

#### By clonal complex

Bacteremic meningococcal pneumonia, abdominal presentations and meningococcal arthritis were all significantly associated with clonal complex 11 (*p* < 0.0001). Bacteremic meningococcal pneumonia was also significantly associated with clonal complex 23 (*p* < 0.0001) (Fig. 4).

Abdominal presentations caused by cc11 isolates dropped over the study period from a maximum of 17 cases in 2017 to only 3 and 4 cases in 2021 and 2022 respectively, whereas other clonal complexes related abdominal presentations and especially the newly defined cc9316 rose sharply in 2022 (Supplementary Fig. 1).

### Early mortality rates of IMD clinical presentations between 2015 and 2022

Early IMD mortality, as defined in the Methods section, was 7.6% and varied non-significantly between 6.25% in 2022 to 8.8% in 2015.

**Fig. 5:**
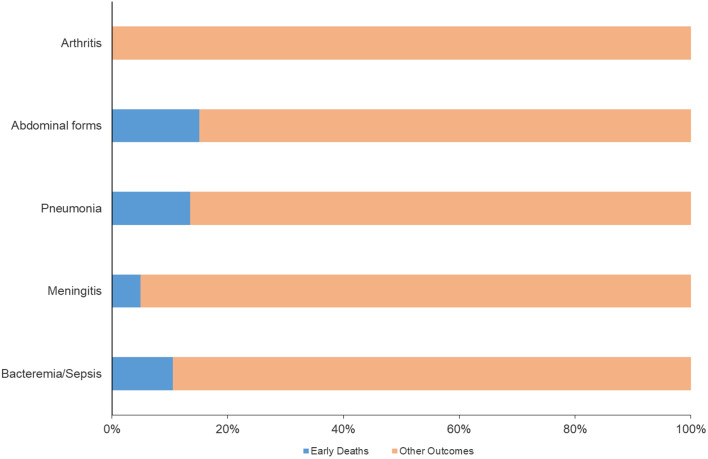
Distribution of early deaths by IMD clinical presentation, France, 2015–2022

Meningeal and arthritis presentations were significantly associated with fewer early deaths (*p* = 0.001 and *p* = 0.0002 respectively). Particularly, there was no death between 2015 and 2022 linked to meningococcal arthritis. Conversely, bacteremic meningococcal pneumonia and abdominal presentations were significantly linked with more early deaths (*p* = 0.0015 and *p* < 0.0001 respectively), with an early mortality rate of 13.5% and 15.1% respectively. Bacteremia/sepsis was also significantly associated with more early deaths (*p* < 0.0001) (Fig. 5).

### Characteristics of serogroup B IMD clinical presentations between 2015 and 2022

IMD due to serogroup B (IMDB) isolates remained the most prevalent (*n* = 1354) between 2015 and 2022, representing an average of half of IMD cases.

Typical presentations (bacteremia/sepsis and meningeal) represented an average of 93% of all IMDB forms. However, the proportion of abdominal presentations significantly increased from 4 to 7.8% in 2022 (*p* = 0.0006) (Fig. 2).

**Fig. 6:**
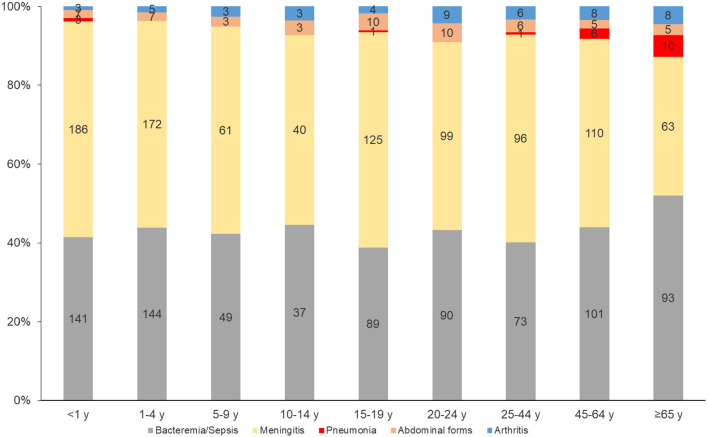
Distribution of serogroup B clinical presentations by age group, France, 2015–2022. (number of cases are indicated in the bars)

IMDB bacteremic meningococcal pneumonia presentation was significantly more prevalent among the 65 and older age group (*p* < 0.0001) (Fig. 6). But no IMDB clinical presentation was significantly associated with a specific clonal complex.

### Characteristics of serogroup C IMD clinical presentations between 2015 and 2022

IMD due to isolates of serogroup C (IMDC) cases evolved in a particular way before the COVID-19 pandemic, due to multiple factors: mainly the modification of the vaccination recommendations against MenC in 2017, the mandatory vaccination in 2018, and the modification of the circulating isolates [14]. The proportion of IMDC cases drastically decreased from 27% of all cases in 2015 to 12.5% in 2019 and then further to 3% in 2021 and 2022, with a very marginal rebound that year [12].

Typical presentations (Bacteremia/sepsis and meningeal) also represented an average of 86% all IMDC forms during the study period. This proportion did not significantly vary but the low number of cases makes the statistical analysis less relevant (Fig. 2).

**Fig. 7:**
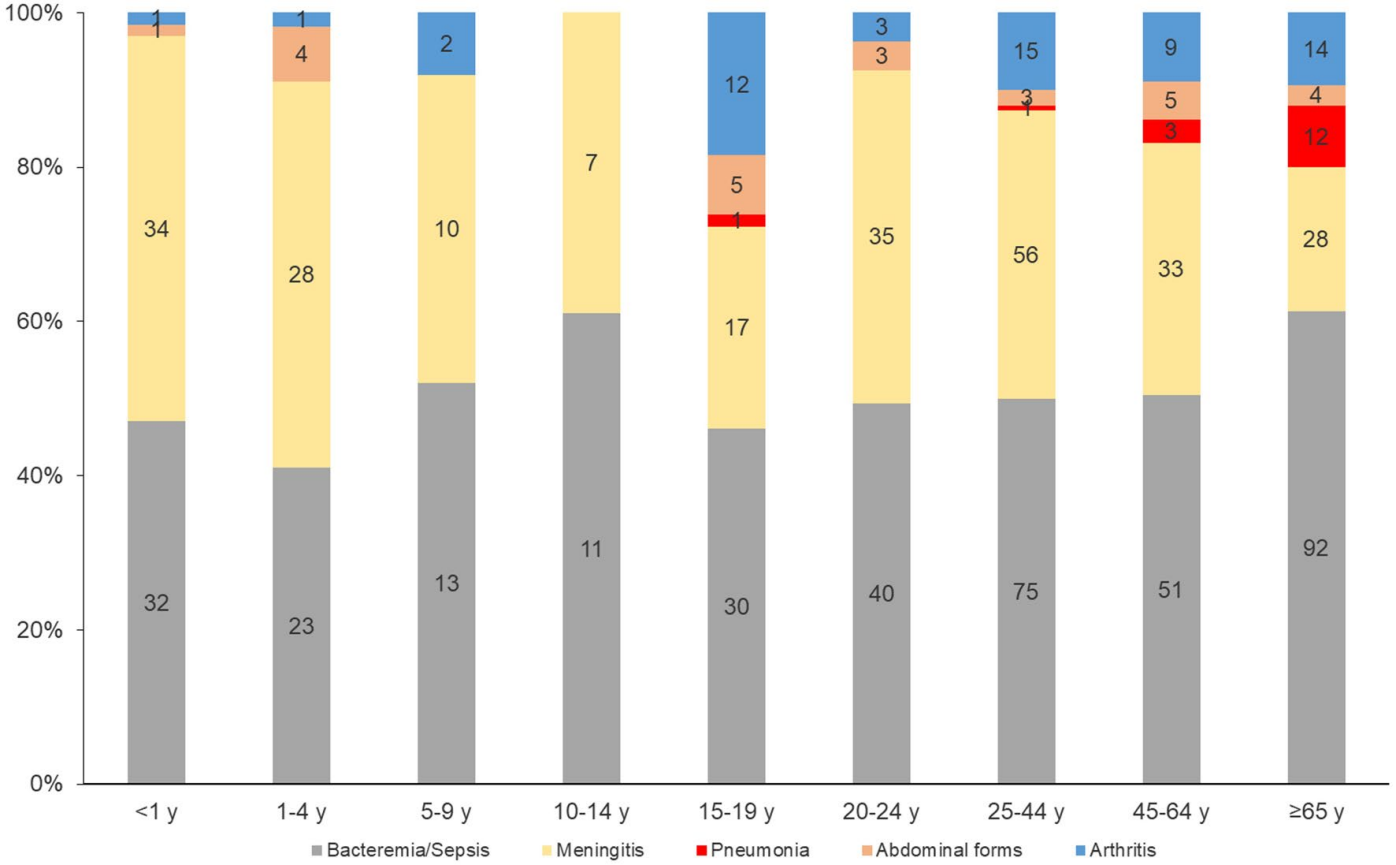
Distribution of serogroup C clinical presentations by age group, France, 2015–2022 (number of cases are indicated in the bars)

When all IMDC were considered, bacteremic pneumonias were significantly more prevalent among the 65 and over age group (*p* < 0.0001) (Fig. 7). Clonal complex 11 represented 91% of identified IMDC cases across the study period but clinical presentations associated with this clonal complex did not vary significantly when compared with the other clonal complexes.

### Characteristics of serogroup W IMD clinical presentations between 2015 and 2022

IMD due to serogroup W isolates (IMDW) have been increasing since 2015 in France, modifying their clinical presentations. Serogroup W was identified in 35% of all IMD abdominal presentations between 2015 and 2022, compared with only 16% between 1991 and 2016 [7].

IMDW decreased during the pandemic but rebounded sharply since 2022 across all age groups, exceeding pre-pandemic levels [12]. However, there was no significant variation in the distribution of clinical presentations from one year to another between 2015 and 2022 (data not shown).

IMDW significantly varied from one age group to another since 2015 (Fig. 2).

**Fig. 8:**
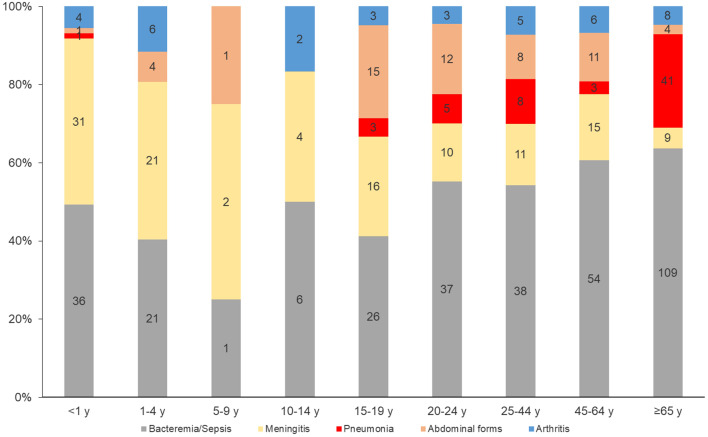
Distribution of serogroup W clinical presentations by age group, France, 2015–2022. (number of cases are indicated in the bars)

Meningeal presentations were significantly more prevalent among the less than 1 year of age (*p* < 0.0001) and the 1–4-year-olds (*p* = 0.0035). Abdominal presentations were significantly more present among the 15–19-year-olds and 20-24-years-olds (*p* = 0.0007). Bacteremic pneumonia presentations were again significantly more prevailing among the 65 and older age group (*p* < 0.0001) (Fig. 8).

As for serogroup C, the hyperinvasive clonal complex 11 represented 63% of all IMDW cases during the study period. But when compared with the other clonal complexes, it did not seem to be associated with a particular clinical form.

### Characteristics of serogroup Y IMD clinical presentations between 2015 and 2022

Unlike the other serogroups, IMD due to serogroup Y isolates (IMDY) were uncommon before the age of 15 and occurred mainly among the 65 and older. But since the COVID-19 pandemic, IMDY increased from 12% in 2019 and 2020 to 25% in 2022, becoming the second most prevalent serogroup in France and rebounding even across younger age groups that year [12].

Despite this sharp rebound, IMDY clinical presentations did not vary significantly on the studied period from one year to another.

**Fig. 9:**
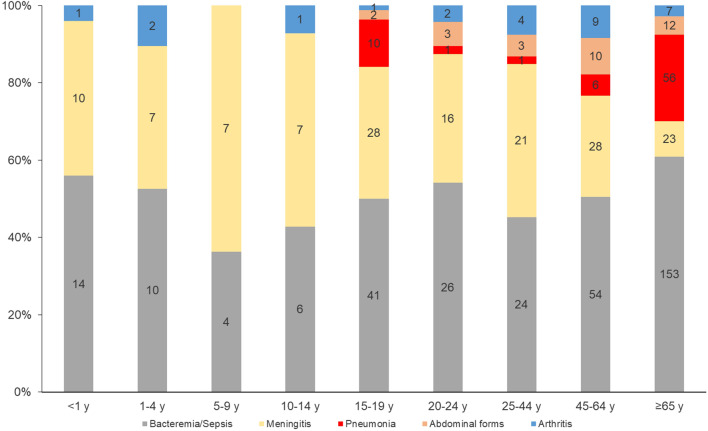
Distribution of serogroup Y clinical presentations by age group, France, 2015–2022. (number of cases are indicated in the bars)

Bacteremic meningococcal pneumonia due to serogroup Y was significantly more prevalent among the 65 and older year-olds (*p* < 0.0001) (Fig. 9). Serogroup Y was associated with 42% of all bacteremic pneumonia presentations between 2015 and 2022 and 46% of bacteremic pneumonia presentations among the 65 and older age group (Figs. 1 and 9).

Clonal complex 23 represented 70% of all IMDY during the study period. But it was not associated with a specific clinical form when compared with the other clonal complexes of serogroup Y (Fig. 4).

## Discussion

The NPI taken to control the COVID-19 pandemic and their lifting since the end of 2022 have led to a sharp rebound in the number of IMD associated with significative phenotypic and genotypic changes [12]. Here we extend this profound impact of the pandemic by reporting the modifications in the spectrum of the clinical manifestations of IMD. Indeed, there was a significant increase in atypical presentations linked to serogroups W and Y that surged at the end of 2022, and even serogroup B cases, that used to manifest almost always by classic presentations, showed an increase in these atypical presentations. The rebound of IMD cases was reported in several countries but few published data on the evolution of clinical forms with mainly case reports and our recent work on the increase of meningococcal epiglottitis in adults [18, 19]. Our data also suggest a shift of IMD disease to older age groups. In particular, bacteremic meningococcal pneumonia was significantly more prevalent among the 65 years and older age group. Abdominal presentations were significantly more prevalent among the 15 to 24 years of age.

These atypical presentations were significantly linked to a higher early mortality, increasing the burden of the disease. Moreover, they can also be misleading and overlooked. For example, the localization of abdominal pain during IMD is frequently around the right iliac fossa that could invoke “acute abdomen” [20–23], leading to unjustified abdominal surgery for an acute appendicitis suspicion [7].

This high number of atypical presentations was informative for abdominal forms in 2022 (*n* = 38, Fig. 1) because it happened despite the significant drop of cc11 isolates that year [12], to which abdominal forms were usually linked [7]. Conversely, non-cc11 abdominal forms represented the majority of cases in 2022 (Supplementary Fig. 1). This suggests that these abdominal presentations which were mostly linked to isolates derived from the South America-UK W/cc11 strain before the COVID-19 pandemic are “shifting” to other genotypes and especially the newly emerging clonal complexes like cc9316 for serogroup W [12].

The emergence of those new genotypes may have been potentiated by an “immunity gap” in the herd immunity of the population during the NPIs, due to the reduced circulation of historical hyperinvasive isolates [12, 24]. Indeed, meningococcal isolates circulated at low levels during the COVID-19 pandemic [25]. Similar low circulation of *Haemophilus influenzae*, *Streptococcus pneumoniae* and influenza A virus was reported among COVID-19 patients in France in 2020 and 2021, suggesting a modification of the nasopharyngeal microbiota [25]. Moreover, non-hyperinvasive clonal complexes are more frequent in carriage and should be expected to recolonize rapidly the nasopharynx and can still be involved in IMD cases [4].

Up to a quarter of patients infected by *SARS-CoV 2* had co-occurring respiratory infections [26], suggesting changes in the upper respiratory microbiota [27]. Moreover, carriage and spread of *N. meningitidis* can be influenced by viral infections [28, 29], but also by other nasopharyngeal bacteria [30]. Changes in nasopharyngeal microbiota may influence its colonization and immune response and subsequently influence the clinical presentation of respiratory pathogens [31].

## Conclusion

The evolution of IMD cases underwent several major changes in France in recent years. But it was the COVID-19 pandemic and the NPI taken to control it that brought about the most profound epidemiological and also clinical changes towards atypical presentations that may delay diagnosis and management. In addition to the rebound in number of IMD cases since the second half of 2022, there was a surge of atypical forms linked to increasing serogroups W and Y in France. IMD in older adults showed a shift to bacteremic meningococcal pneumonia and young adults to abdominal presentations. These shifts was associated with an increasing early mortality of IMD.

Continued monitoring and analysis of the epidemiological and clinical changes of IMDs are therefore crucial now that the NPI are almost lifted across the world and with scheduled mass gatherings for 2024 in France (Olympic Games) and over the world (several pilgrimages and world youth day) which can facilitate the spread of cases [32]. The recent alert on IMDW cases linked to recent travel to Saudia Arabia needs to enhance surveillance and vaccination use [33].

### Electronic supplementary material

Below is the link to the electronic supplementary material.

Supplementary Material 1

## Data Availability

Sequencing data have been published in a previous study [12] and are available on https://pubmlst.org/organisms/neisseria-spp upon filtering on country (France) and by year (2015-2022). Other data that support the findings of this study are available on request from the corresponding author [ST].
