# Supplementary figures and images for: Recent increase in atypical presentations of invasive meningococcal disease in France
Source: BMC Infect Dis. 2024 Jun 26;24:640. doi: 10.1186/s12879-024-09547-y (PMC11200843; doi:10.1186/s12879-024-09547-y)

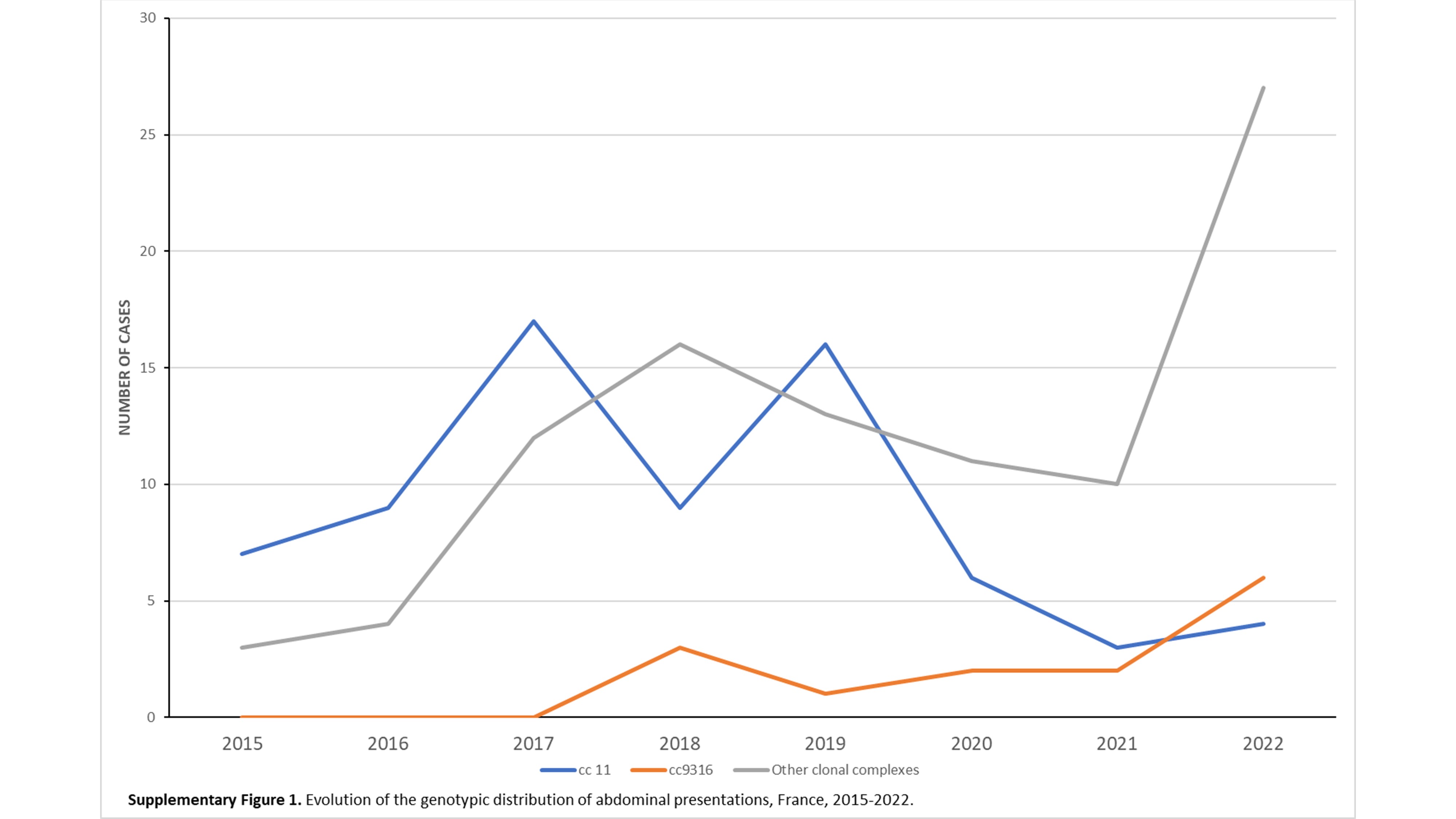

Supplement: Supplementary file 1 — Supplementary Material 1 [file 12879_2024_9547_MOESM1_ESM.jpg]
